# Dysfunctional Heteroreceptor Complexes as Novel Targets for the Treatment of Major Depressive and Anxiety Disorders

**DOI:** 10.3390/cells11111826

**Published:** 2022-06-02

**Authors:** Miguel Pérez de la Mora, Dasiel O. Borroto-Escuela, Minerva Crespo-Ramírez, José del Carmen Rejón-Orantes, Daniel Alejandro Palacios-Lagunas, Magda K. Martínez-Mata, Daniela Sánchez-Luna, Emiliano Tesoro-Cruz, Kjell Fuxe

**Affiliations:** 1 Instituto de Fisiología Celular, Universidad Nacional Autónoma de México, Mexico City 04510, Mexico; mcrespo@ifc.unam.mx (M.C.-R.); dpalacios@ifc.unam.mx (D.A.P.-L.); magdamartinez@ifc.unam.mx (M.K.M.-M.); dsanchez@ifc.unam.mx (D.S.-L.); 2 Department of Neuroscience, Karolinska Institutet, Biomedicum, Solnavägen 9., 17177 Stockholm, Sweden; Dasiel.Borroto.Escuela@ki.se (D.O.B.-E.); kjell.fuxe@ki.se (K.F.); 3 Department of Biomolecular Science, Section of Morphology, Physiology and Environmental Biology, University of Urbino, Campus Scientifico Enrico Mattei, via Ca’ le Suore 2, I-61029 Urbino, Italy; 4 Facultad de Medicina, Instituto de Investigación Biomédica de Málaga, Universidad de Málaga, Campus Teatinos S/N., 29071 Málaga, Spain; 5 Laboratorio Experimental de Farmacobiología, Facultad de Medicina Humana C-II, Universidad Autónoma de Chiapas, Tuxtla Gutiérrez 29026, Mexico; jose.rejon@unach.mx; 6 Unidad de Investigación Biomédica en Inmunología e Infectología, Hospital de Infectología, Centro Médico Nacional La Raza, IMSS, Mexico City 01000, Mexico; emiliano_tesoro@hotmail.com

**Keywords:** G-protein coupled receptors, receptor-receptor interactions, heteromeric complexes, receptor oligomerization, anxiety, depression

## Abstract

Among mental diseases, major depressive disorder (MDD) and anxiety deserve a special place due to their high prevalence and their negative impact both on society and patients suffering from these disorders. Consequently, the development of novel strategies designed to treat them quickly and efficiently, without or at least having limited side effects, is considered a highly important goal. Growing evidence indicates that emerging properties are developed on recognition, trafficking, and signaling of G-protein coupled receptors (GPCRs) upon their heteromerization with other types of GPCRs, receptor tyrosine kinases, and ionotropic receptors such as N-methyl-D-aspartate (NMDA) receptors. Therefore, to develop new treatments for MDD and anxiety, it will be important to identify the most vulnerable heteroreceptor complexes involved in MDD and anxiety. This review focuses on how GPCRs, especially serotonin, dopamine, galanin, and opioid heteroreceptor complexes, modulate synaptic and volume transmission in the limbic networks of the brain. We attempt to provide information showing how these emerging concepts can contribute to finding new ways to treat both MDD and anxiety disorders.

## 1. Introduction

Converging evidence has shown that imbalances among different homo and hetero neurotransmitter receptor complexes; have a critical contribution to both the pathophysiology and the clinical course of psychiatric disorders [1,2,3]. Efforts to influence such abnormal imbalances may be of value for their proper and successful treatment. Therefore, in this paper, after assessing the epidemiological aspects concerning depression and anxiety and examining some of the basic principles that govern and characterize receptor oligomerization, we will review most of what has been gathered on the role of homo/heteromers in mood and emotion. We will also refer here to some new therapeutic strategies based on what is known about receptor oligomerization to treat or at least mitigate either MDD or anxiety disorders. As a word of caution, although different clinical forms of (either anxiety or depression disorders) do exist, in this review, no attempts will be usually made to link any described homo/heteromer with any of them.

## 2. Clinical Characteristics and Epidemiological Aspects Related to Depression and Anxiety

### 2.1. Major Depressive Disorder

The definition of major depressive disorder (MDD) is based on several symptoms that form a syndrome that causes functional impairment. The two main classificatory diagnostic systems are the Diagnostic and Statistical Manual of Mental Disorders (DSM), published by the American Psychiatric Association (APA), and the International Classification of Diseases (ICD), published by the World Health Organization (WHO) [4]. These diagnostic systems are based on identifying several key symptoms that are present in most depressive patients. The most specific symptoms of a depressive disorder are a decreased ability to experience pleasure (anhedonia) and its diurnal variation, which intensifies the guilt of being ill [5].

MDD is a significant health problem due to its high prevalence, negative impact on quality of life, and its association with suicide. In the 1990s, the WHO ranked depression as the fourth leading cause of disability [6] and it is expected that by 2030 it will occupy the first position [5,7,8]. The lifetime prevalence of MDD varies considerably among countries in such a way that its 12-month prevalence ranges from 0.3% (Czech Republic) to 10.0% (USA), with midpoints at 4.5% (Mexico) and 5.2% (Germany) [7,9,10,11].

### 2.2. Anxiety

Anxiety can be defined as a psychiatric disorder characterized by an excessive fear of a threat that does not actually correspond to a real threat, such that the response is not proportional to the actual risk or danger posed. It is of vital importance to differentiate physiological anxiety from pathological anxiety. The former occurs due to the perception of a real and imminent threat, while pathological anxiety is a state of anticipation of the perception of future threats [12].

Anxiety disorders involve dysfunction in brain circuits that respond to danger. Pathological anxiety is characterized by excessive fear or disproportionate reaction to posed threats. On the other hand, anxiety disorders are among the most prevalent and disabling mental health pathologies worldwide [13,14]. One in ten people suffers from anxiety or panic disorders. Approximately 20% to 30% of adolescents and young adults are affected by anxiety disorders [15] and commonly develop a generalized anxiety disorder at the beginning of their adulthood [16]. The prevalence of symptoms of post-traumatic stress disorder (PTSD) increased during the recent outbreak of infectious diseases during the COVID-19 pandemic, affecting people’s physical and mental health worldwide [17]. The Frequency of anxiety is twice as high in women as in men [15] and its prevalence, as occurs with depression, varies widely from country to country. Lifetime prevalence is higher in high-income countries compared to low-income ones. Based on surveys, up to 33.7% of the population is affected by an anxiety disorder during their lifetime [18]. World health surveys report averages of 3.7% and 1.8% in a lifetime and 12-month prevalence, respectively [19].

### 2.3. Clinical Responses Associated to the Use of Pharmacological Compounds in Patients Suffering from Depression and Anxiety Disorders

Many compounds have been developed to treat depression and anxiety, but in many cases, their use has been curtailed by the appearance of numerous side effects. In addition, considerable pharmacodynamic differences have been found between antidepressants and anxiolytic drugs. Thus, while acute anxiety signs and symptoms subdue in a matter of minutes/hours following treatment [20], antidepressants require several weeks or even months to be clinically effective. Furthermore, as it will also be considered below (see Section 6.1), a significant proportion of depressed [21,22] and anxious [23,24,25] patients fail to achieve sustained remission. In view of the above, and as depression and anxiety represents a major public health problem, novel strategies such as targeting G protein-coupled receptors (GPCRs) dimers/oligomers the promise of developing new and more finely tuned therapeutics to treat both anxiety and particularly affective disorders.

## 3. Emergent Concepts of Receptor Heterodimerization and Their Impact on Nervous System Function

From the original proposal of Kjell Fuxe and Luigi Agnati in the early 1980s [26,27,28], accumulating evidence has led to the current view that GPCRs do not only exist and act as individual molecular entities, as was previously demonstrated [29,30], but can also exist as homo and heteroreceptor complexes, with or without the aid of adapter or scaffold proteins [3], to form homo and heterodimers or even higher-order receptor oligomers in which a variety of receptors a particular stoichiometry, composition and topography are present (for reviews see: [28,31,32,33,34,35,36,37,38,39,40,41,42,43]).

As receptor homo and heteromerization seems to be, in most cases, formed by the cumulative actions of numerous weak binding forces such as hydrogen bridges, hydrophobic forces, and salt-type interactions, many have cast doubts on the actual existence of receptor oligomers. The discovery that a covalent association between GABAB1 and GABAB2 receptor subunits was required to form an active GABAB receptor heterodimer was of great importance [37,39,44]. It gave strong support to the receptor homo and heteromerization concept. In support of this notion, X-ray crystallographic studies carried out in three different crystal structures of the extracellular ligand-binding region of mGluR1 showed the existence of disulfide-linked homodimers [45].

**Figure 1:**
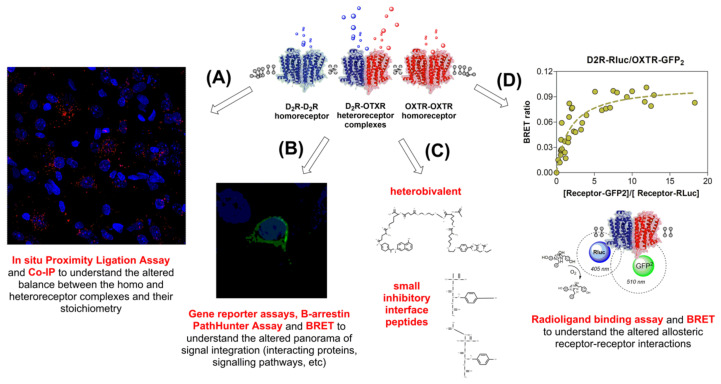
Several biophysical (BRET, FRET) and biochemical (radioligand binding assay, in situ Proximity Ligation Assay (in situ PLA), Gene Reporter Assay, Co-immunoprecipitation (CO-IP), and internalization assays (Path-hunter arrestin)) methods have made possible the understanding of the molecular mechanisms involved in the integration of the receptor-receptor interactions in the plasma membrane. Herein (central panel), the example of the dopamine (D2R) and Oxytocin (OXTR) homo and heteroreceptor complexes and their balance. (**A**) Illustration of the in situ PLA analysis of the D2R-OXTR heteroreceptor complexes (red blub/clusters) in the nucleus accumbens of the rat brain. (**B**) Gene reporter assays (CREB, SRE, NFAT, etc.) and internalization assays (B-arrestin Path-hunter assay, etc.) made possible the understanding of the panorama of the signal integration and transcriptional machinery after the activation of the various homo and heteroreceptor complexes in the plasma membrane. (**C**) The developments of heterobivalent and small interface peptides made it possible to study the receptor-receptor interface of these complexes. (**D**) Illustration of the analysis of the formation of D2R-OXTR heteroreceptor complexes using Bioluminescence resonance energy transfer method (BRET2).

Receptor homo and heteromerization have been proven to be an important principle within the GPCRs realm. Numerous powerful pharmacological, biochemical, and biophysical techniques have been successfully applied for this purpose. Thus, apart from the classical binding studies and the immunoprecipitation procedures followed by Western blot analysis, both cross-linking studies in which receptor dimers are selectively taken (i.e., Paglin and Jaimeson, 1982) [46] and co-immunoprecipitation using antibodies to epitope-tagged receptors [47] have been alternatively used to this end. More recently, biophysical techniques such as fluorescence resonance energy transfer (FRET) [48,49], bioluminescence resonance energy transfer (BRET) [49,50,51], the combination of both [52,53] and the proximity ligation assay (PLA) [54,55,56,57] have been introduced to overcome many of the inherent limitations of the previously used methodology (Figure 1).

Three-dimensional structure determination of GPCRs oligomers due to their conformational flexibility, which undermines their stability, has been a tremendous challenge that has only been achieved to a very limited extent see [45]. However, it is expected that emerging approaches [58], such as serial femtosecond crystallography, micro electron diffraction, and single-particle electron cryo-microscopy, will allow us to have at least an insight into their three-dimensional structure.

By applying these new methods, it is now known that as a consequence of the physical interaction between GPCRs receptors (protomers), allosteric changes are established in each of them [49,59], leading to the development of emergent properties involving changes in ligand recognition, trafficking, and signaling, which are different to those originally present in the interacting protomers (Figure 1) [31,49,60,61,62,63,64]. Furthermore, it has also been discovered that receptor homo and heteromerization are not only limited to GPCRs interactions but also exist between GPCRs and ionotropic receptors, like GABA and glutamate receptors [40,65,66,67,68] and also with GIRK channels [69] and neurotransmitter transporters [70,71]. In addition, GPCR interactions with tyrosine kinase receptor (RTK) proteins, giving them a role also in trophic function [3,72,73,74,75], have also been demonstrated.

Based on the foregoing discussion, it is evident that receptor homo-and heteromerization gives a glimpse of a previously unexpected view where either a physiological or pharmacological action acting at a GPCRs belonging to a particular heteroreceptor complex can influence and change the activity of its (their) protomer partner(s) in terms of agonist/antagonist receptor recognition, receptor trafficking, and receptor signaling activities. It opens the possibility of generating new pharmacology and developing new therapeutic approaches. Taken together, it leads to a new concept on how GPCRs can modulate synaptic and volume transmission, including neural plasticity and learning and memory.

## 4. Receptor Homo and Heterodimerization with Relevance for MDD

MDD, as indicated at the beginning of this review, is a multifactorial disorder that refers to several discrete clinical entities collectively characterized by the presence of anhedonia and in which a role for several kinds of heteroreceptor complexes have been identified. As 5-HT, DA, and NA neurotransmission have been proved to have a paramount importance for this disorder, we will to a substantial degree, concentrate in this review on their mutual receptor-receptor interactions and their interactions with other types of receptor systems. Likewise, as there exists an important comorbidity between MDD and anxiety [76,77], we will continuously refer to this association both in this and in the following sections of this paper.

### 4.1. Role of 5-HT Transporters and Receptor Systems in MDD

As a result of the collaboration carried out by Kjell Fuxe and the Nobel laureate Arvid Carlsson in the late 1960s to the early 1970s, the basis of the serotonin theory of MDD was built up, allowing numerous insights into several aspects of this pathological condition, including the development of selective serotonin reuptake inhibitors (SSRIs), which have been proven to be effective to treat different clinical forms of MDD. Thus, evidence was obtained indicating for the first time the existence of 5-HT uptake mechanisms in serotonin neurons [78] and that imipramine and amitriptyline, considered at that time as relatively good antidepressant drugs, could reduce 5-HT uptake [79].

**Figure 2:**
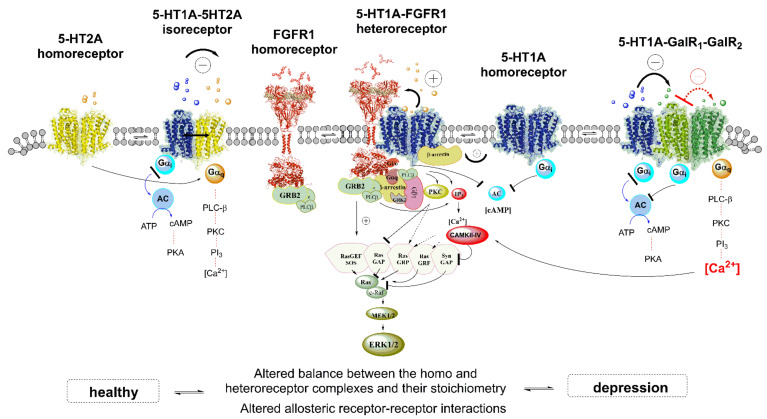
Panorama of serotonin homo and heteroreceptor complexes of relevance for Major Depressive Disorders (MDD) with a location in the plasma membrane. Recent work has strongly indicated the impact of 5-HT1AR-5-HT2AR isoreceptor complexes and the 5-HT1AR-FGFR1, 5-HT1AR-GalR1-GalR2 heteroreceptor complexes and their balance between each other in MDD. The significant molecular mechanism is represented by allosteric receptor-receptor interactions within the heteroreceptor complexes. Inhibitory and enhancing allosteric receptor-receptor interactions develop in the 5-HT1AR-5HT2AR and 5-HT1AR-FGFR1 heteroreceptor complexes, respectively. In the trimeric 5-HT1AR-GalR1-GalR2 heteroreceptor complexes, a cascade of inhibitory interactions takes place. The 5-HT1AR protomer produced a negative allosteric modulation of the GalR1 protomer, inducing a removal of the inhibitory allosteric interactions between the GalR1 and GalR2 protomers (indicated as red dashed lines), leading to antidepressant events. Another molecular mechanism of strong interest is the potential disbalance that may develop between the various types of homo and heteroreceptor complexes. These molecular mechanisms involve both changes in receptor protomers recognition, signaling (including the panorama of interacting proteins and the intracellular cascades), and internalization. These events take place in key neuronal pathways of the limbic system, having an important role in emotional events.

Today, numerous 5-HT receptor subtypes have been identified in the mammalian brain. Of these receptors, only one of them (5-HT_3_) qualifies as an ionotropic receptor, whereas the other 5-HT receptors (5-HTR) are GPCRs (5-HT1R, 5-HT2R, 5-HT4R, 5-HT5R, 5-HT6R, 5-HT7R). They are assembled into six different 5-HTR families [80]. Certain 5-HTR subtypes can interact either with other types of 5-HTR or with other receptors not belonging to the 5-HTR family. Furthermore, recent findings have revealed that 5-HTR heterocomplexes are also in balance with the corresponding homomers, including the monomers [81] (Figure 2).

#### 4.1.1. Role of 5-HT1AR-5-HT2AR Heteroreceptor Complexes in MDD

Evidence for the existence of 5-HT1aR-5-HT2AR heterocomplexes in the brain has been gathered within the dorsal hippocampus and the anterior cingulate cortex of the rat [73], which are well known for their important participation in the biology of MDD [82,83]. These heteroreceptor complexes are also known as isoreceptor complexes as the two receptor protomers are activated by the same transmitter. 5-HT also is named serotonin. The existence of this heterocomplex was validated [73] by BRET in HEK293T cells. In turn, functional studies demonstrated antagonistic allosteric receptor-receptor interactions between the two 5-HT protomers, whereby agonist activated 5-HT2AR reduced the affinity of the 5-HT1AR protomer for ipsapirone, a 5-HT1AR partial agonist [73]) (Figure 2). In view of these results, it is of considerable interest that in mice previously exposed to a post-traumatic stress model, treatment with WAY100635, a 5-HT1AR antagonist, enhances the expression of 5-HT2AR and at the same time reduces the 5-HT1AR expression within the hippocampus [84] These results support the existence of antagonistic interactions between the 5-HT1aR and 5-HT2AR protomers in the hippocampus and indicate that the 5-HT2AR mediated inhibition of the 5-HT1a receptor protomer can be involved in producing post-traumatic stress.

Additionally, it is rather interesting that a reduction in the density of PLA positive clusters was also found in the CA1 and CA2 regions of the hippocampus 2 h after the exposure of the rats to the Forced Swim Test [73], pointing to a likely rapid participation of the 5-HT1AR-5-HT2AR heterocomplexes in the pathophysiology of MDD. Thus, considering that an elevated 5-HT1AR activity is associated with antidepressant effects [85] and that a depressant-like behavior is linked to an enhancement of 5-HT2AR activity [86,87,88], it was proposed by Borroto-Escuela et al. [73] that heterobivalent drugs having both 5-HT1AR agonistic and 5-HT2AR antagonistic pharmacophores may be useful for the treatment of MDD.

It is of particular interest to understand how these 5-HT heteroreceptor complexes modulate synaptic and volume transmission in the limbic system of the brain. 5-HT mainly operates via short distance (µm range) volume transmission in the extracellular space of the brain to activate various 5-HTR subtypes located on 5-HT and other types of neurotransmitter terminals, e.g., the limbic system [89,90] 5-HT terminal networks also exist on the ependymal cells along the ventricles, with released 5-HT flowing via volume transmission in the cerebrospinal fluid before reaching their 5-HTR targets via diffusion and flow along with the perivascular networks [91].

A highly significant majority of the different types of 5-HT heteroreceptor complexes are located extrasynaptically in the plasma membrane of the limbic system [91,92,93]. Thus, these complexes are mainly modulated by volume transmission. However, 5-HT heteroreceptor complexes also exist in the 5-HT synapses, where they can modulate synaptic 5-HT transmission in the pre- and postsynaptic membranes. In addition, synaptic 5-HT heteroreceptor complexes likely exist also in other types of neurochemical synapses like glutamate and GABA synapses to participate in learning and memory, where also emotions can play a significant role [89,94,95,96].

Future relevance for MDD. 5-HT1aR-5-HT2AR heteroreceptor complex is of particular interest as the 5-HT1aR protomer, when activated, produces antidepressant actions while the 5-HT2AR protomer, when activated, causes depressive effects. Therefore, this complex represents an exciting integrative structure of high relevance for MDD in which the bidirectional allosteric receptor-receptor interactions should be fully characterized. It will be of high interest to test the vulnerability of this 5-HT1AR-5-HT2AR complex in animal models of MDD.

#### 4.1.2. 5-HT1AR-FGFR1 Heteroreceptor Complex in MDD

It has been demonstrated by PLA analysis that 5-HT1AR-FGFR1 interactions take place in neurons localized both in rat dorsal hippocampus and midbrain raphe in somato-dendritic serotonin neurons enriched in 5-HT1AR autoreceptors [74,85,97,98,99] (Figure 2). The positive interactions lead to increased FGFR1-mediated plasticity, which correlates with antidepressant activity, as measured in the Forced Swim Test. [100] Interestingly, it was also shown [101] that agonist-induced activation of FGFR1 resulted in a reduction of the 5-HT1AR-induced opening of GIRK channels [102] in 5-HT1AR positive pyramidal glutamate nerve cell bodies in the CA1 region of the dorsal hippocampus [101]. So, it is likely that within the 5-HT1AR-FGFR1 heterocomplexes in the hippocampus and particularly in midbrain raphe neurons, an enhancement of FGFR1 protomer activity following agonist activation can be able to induce conformational changes in the 5-HT1AR protomer impairing its ability to open GIRK channels in these neurons avoiding their hyperpolarization with a possible enhanced firing of especially the dorsal raphe serotonin neurons leading to antidepressant actions. Thus, under these conditions, a reduction in 5-HT1AR signaling may lead to an increase in plasticity and to the development of antidepressant effects. Thus, a new strategy based on the above findings for treating MDD may benefit from any approach aimed to promote the inhibitory allosteric influence of FGFR1 protomers, especially on the 5HT1AR autoreceptor protomer, to reduce its hyperpolarization of the mesencephalic raphe serotonin neurons.

It should be noticed that the 5-HT1AR autoreceptor in the dorsal raphe becomes activated via 5-HT volume transmission through 5-HT release from 5-HT nerve cell bodies and dendrites [91]. An important novelty is that the activation of the 5-HT1AR autoreceptor not only inhibits the firing of the dorsal raphe neurons but also increases trophic activity in the FGFR1 protomer in the 5-HT1A autoreceptor-FGFR1 heterocomplex [89] with counteraction of the atrophy development in the ascending 5-HT neurons. The current results furthermore indicate that, in addition, the activated FGFR1 protomer via allosteric and phosphorylation mechanisms can bring down the ability of the 5-HT1AR autoreceptor protomer to open the GIRK channels and reduce inhibition of the 5-HT neuronal activity in the dorsal raphe neurons. It illustrates the impact of RTK-5-HT1AR heterocomplexes may have in counteracting MDD.

Future relevance for MDD. It will be of high interest to compare different animal models of MDD, including a genetic model [103], the vulnerability of the FGFR1–5-HT1AR heterocomplex, known to be preferentially located in the hippocampal neurons and mesencephalic serotonin neurons. The relevance of the trophic actions vs. reductions of the 5-HT1AR autoreceptor function of these heterocomplexes should be established with regard to antidepressant activity in the models tested.

#### 4.1.3. 5-HT-Galanin Heterocomplexes in MDD

Galanin, referred to here as Gal (1–29), is a 29 amino acid neuropeptide [104], which binds with high affinity to several receptor subtypes designated as GalR1, GalR2, and GalR3 [105,106]. As the pioneering work of Fuxe et al. in 1980 [107] demonstrated that Gal (1–29) could reduce firing in ascending 5-HT neurons, this neuropeptide was considered to elicit important effects on mood regulation [106,108,109,110,111,112,113].

Accumulating evidence has shown that Gal (1–29) has a critical role in the mesencephalic serotonergic neurotransmission [114]. In addition, Gal (1–29) receptors are widely distributed in the brain [115], including some regions having a special involvement in MDD, such as the dorsal raphe [116,117], where the coexistence of Gal (1–29) and 5-HT was demonstrated by Melander et al. in 1986 [118]. GalR1 and GalR3 signal through the inhibitory G-protein Gi/o, whereas GalR2 activates the excitatory G protein G_q/11_ [105].

From the point of view of its behavioral effects on MDD, preclinical studies conducted in rodents have shown that in Flinders sensitive line rats, which are considered to represent a genetic model of depression [119], [^125^I]galanin binding sites are found to be increased in the dorsal raphe nucleus and that Gal (1–29) can activate in a differential way its receptors eliciting either depressant or antidepressant effects depending on which activated 5-HT receptor subtype is in dominance [120,121,122]. Thus, while activation of both GalR1 and GalR3 receptor subtypes elicit depressant effects [112,120,121,123], GalR2 activity has antidepressant properties [108,120,124,125,126]. In line with the above, mice overexpressing Gal2 receptors exhibited a decreased immobility in the Forced Swim Test [[127], which suggests antidepressant effects, whereas Gal3 knockout mice were reported to show a trend toward a shorter immobility time in the tail suspension test [128].

On the other hand, quantitative autoradiographic studies unveiled the existence within the brain of high-affinity binding sites for Gal (1–15) peptide, a Gal (1–29) amino-terminal fragment [31,91,129] having neurochemical effects in the brain [130]. [^125^I]galanin (1–15) binding studies revealed that this Gal (1–29) fragment was bound with a pronounced ability and a marked specificity to recognition sites within the hippocampus, neocortex, and striatum. As these regions display a negligible binding for Gal (1–29) [129], this finding suggests the presence of distinct Gal (1–15) binding sites within these areas. Furthermore, in support of this view, evidence was provided using both Bioluminescence Resonance Energy Transfer (BRET^2^) and PLA analysis that both in HEK293R cells and in brain sections that GalR1 and GalR2 were sufficiently close to each other (≤20 Å) to interact and engage in the formation of GalR1-GalR2 heterocomplexes [2,34,49,122,131]. Thus, based on these studies, it was suggested by Borroto-Escuela et al. [2] that the Gal1R-Gal2R heterocomplex may indeed be the true receptor for Gal (1–15). In support of this suggestion, it was unveiled that following the silencing of some genes involved in the expression of GalR1 and GalR2 by the use of small interference RNAs (siRNA) [132,133], it was possible to interfere with the already demonstrated GalR1 and GalR2 colocalization in both hippocampus and dorsal raphe and most importantly to block the behavioral effects triggered by the i.c.v., administration of Gal (1–15) fragment [109,113].

From the point of view of the functional role of the above newly discovered binding sites, it was demonstrated that Gal (1–15) was able to induce depressant effects in rats [109,131,132,133,134,135], as its i.c.v., administration was followed by an increase in the immobility time that the rats experienced both in the Forced Swim Test and the Tail Suspension Test, as compared with non-treated control animals [109].

On the other hand, compelling experimental evidence has disclosed important pharmacological pre- and post-synaptic interactions between 5-HT and Gal (1–29) systems, which support the existence of 5-HT-Gal (1–29) receptor heteromers and their participation in the modulation of mood and anxiety. Thus, in addition to the indicated coexistence of 5-HT and Gal (1–29)-IR in the dorsal raphe cell bodies [109], it has also been documented by Fuxe’s group [136] that i.c.v. Gal (1–29) injections were able to reduce metabolic and perhaps an associated signaling activity in dorsal raphe-limbic 5-HT targets, most probably due to an inhibitory effect on the ascending 5-HT neurons located in this region [137]. On the other hand, Gal (1–29) was shown to reduce the affinity of the 5-HT1AR agonist [^3^H]8-hydroxy-2-(di-n-propylamino) tetralin ([^3^H]DPAT) binding sites in membranes from the rat ventral limbic cortex. As these membranes are presumably mostly derived from postsynaptic elements, this result suggests that Gal (1–29) can modulate postsynaptic 5-HT1AR signaling in the limbic system [137] through antagonistic interactions elicited within GalR-5-HT1AR heterocomplexes. Conversely, activation of 5-HT1AR in membranes and brain sections from di- and tele-encephalic areas of the rat was shown to increase the affinity of Gal (1–29) receptors [129], indicating, in putative GalR-5-HT1AR heterocomplexes, the existence of reciprocal interactions between the two types of receptor protomers (Figure 2). In addition to the above, it has been observed that Gal (1–15) but not Gal (1–29) can modulate in an antagonistic way 5-HT1AR in the dorsal hippocampus. It was proposed that GalR1-GalR2-5-HT1AR receptor mosaics can exist in the hippocampus, on dorsal raphe cells, and other limbic neurons where galanin fragment 1–15 is also able to exert allosteric interactions via modulation of the GalR1-GalR2 component of the trimeric hetero receptor complex to enhance 5-HT1AR protomer function in the basal state of this heterocomplex [138].

Like all neuropeptide signals, galanin and its fragment Gal (1–15) communicate via short (µm range) and long-distance (µm range) volume transmission in the brain, also involving diffusion and flow along with fiber bundles and blood vessels and in the cerebrospinal fluid. The different types of galanin heteroreceptor complexes are mainly located in extrasynaptic membranes and, therefore, to a high degree, exert their major modulation on volume transmission. However, galanin peptides can also exist in synaptic membranes and can, therefore, also modulate synaptic transmission (see above).

Future relevance for MDD. It will be of high interest to clarify the role of Gal (1–29) vs. the Gal (1–15) as to their role in models of MDD and in modulating GalR1-GalR2-5-HT1AR and GalR1-5-HT1AR heterocomplexes. OBS: are you referring here to GalR1-5HT1AR or GalR1-GalR2 heterocomplexes? Are these heteroreceptor complexes altered and associated with significant changes in the actions of Gal (1–29) and Gal (15)? The entry of Gal (1–29) and Gal (1–15) into the brain should also be compared.

#### 4.1.4. 5-HTR-OXTR Heterocomplexes in Depression

Receptor-receptor interactions involving 5-HTR and oxytocin receptors (OXTR) have been recognized during the last few years. Accordingly, 5-HT2AR-OXTR [139] and 5-HT2CR-OXTR [140] heterocomplexes have been identified and studied in cellular models using flow cytometry-based FRET and PLA combined with confocal microscopy. Their presence [139,140] has also been demonstrated in brain sections using PLA. Interestingly, although both types of heterocomplexes were found to be widespread in the brain, they are particularly abundant within limbic regions (hippocampus, cingulate cortex, and nucleus accumbens), suggesting that they may play a prominent role in mood, cognition, anxiety, and social behavior [141]. Although the functional consequences of these interactions for brain function are largely unknown, antagonistic allosteric receptor-receptor interactions have been disclosed using living HEK 293 cells. It includes both 5-HT2AR–OXTR and 5-HT2C-OXTR heterocomplexes [139,140], as well as 5-HT2AR-5-HT2CR and 5-HT2BR-5-HT2CR isoreceptor complexes in which the 5-HT2C protomer allosterically strongly inhibits the 5-HT2AR and 5-HT2BR protomer signaling [142]. These results are of considerable interest for understanding the functional role of these 5-HTR heteroreceptor complexes. Observations exist that in most cases, the 5-HT2CR protomer and the 5-HT2AR protomer, both forming heterocomplexes with the OXTR protomer, exert a dominant inhibitory influence on the OXTR protomer, significantly blunting its signaling. In line with these results, an inactive form of the 5-HT2CR in the locus coeruleus NA nerve cells leads to a decrease in noradrenergic neurotransmission in the locus coeruleus, which is dependent on 5-HT2AR activity [142]. It should also be considered that the OXTR can mediate antipsychotic effects. It may also have a role in social and emotional behaviors in rodents and humans, which includes bonding and trust through regulation of the limbic circuits [143].

It is proposed that in vivo activation of depressive and anxiogenic 5-HT2AR and 5-HT2CR [109,144] within their corresponding heteromeric complexes leads to a reduction of OXTR protomer-mediated neurotransmission. OXTR activity has been proven to have beneficial effects on mood [145,146,147]. Therefore, it is proposed that its blunting can result in the appearance of depressive behavior. It will remain for future experiments to test this hypothesis.

Oxytocin is a well-known hormone and communicates via short-and and long-distance volume transmission in the brain. Therefore, the OXTR heteroreceptor complexes should mainly exist in extrasynaptic membranes and preferentially modulate volume transmission. However, their presence in synaptic membranes cannot be excluded to allow an integrative role of OXTR heterocomplexes in synaptic transmission.

Future relevance for MDD. These significant results are of considerable interest. The OXTR-5-HT2AR and especially OXTR-5-HT2CR receptor hetercomplexes should be studied in the limbic circuits in models of MDD. It will give indications if especially 5-HT2CR protomer is a major factor in mediating MDD involving a strong blockade of the OXTR function. Can differential effects be observed in the different models tested?

#### 4.1.5. 5-HT1AR-D2_L_R Heterocomplexes in MDD

Evidence for the existence of D2_L_R-5-HT1AR heterocomplexes has been gathered by both Lukasiewicz et al. 2016 [148] and Shioda et al. 2019 [149] in cellular models and in the dorsal raphe nucleus [149]. In addition, the presence of this heteroreceptor complex within the hippocampus and the coexistence of D2R and 5-HT1AR in cells of this area was immunohistochemically validated. Its presence was ascertained by both PLA and immunoblotting experiments carried out in extracts from this nucleus [149]. Functionally it was shown that lack of the dopamine D2_L_R isoform in D2_L_R KO mice caused stress vulnerability. It was explained by the absence of the feedback exerted by the D2_L_R-5HT1AR heterocomplex on 5-HT release mediated by GIRK channel activation [149].

DA and 5-HT mainly operate via volume transmission but can also include synaptic transmission. In agreement, the 5-HT1AR-D2_L_R heterocomplexes are mainly found in extrasynaptic membranes. Therefore, the 5-HT1AR-D2_L_ heterocomplexes mainly modulate volume transmission. However, it seems likely that they also can participate in synaptic transmission in view of their likely existence also in synaptic membranes.

Future relevance for MDD. These results open the existence of a new type of 5-HTR-DAR heterocomplex based on the demonstration of D2_L_R-5-HT1AR heterocomplexes in the limbic system. It will be of substantial relevance to test if this complex is vulnerable to changes in models of MDD. Furthermore, the changes should be compared to those potentially obtained in D2R-5-HT2AR heterocomplexes in these experiments [99,150]. These studies can give an increased understanding of the impact of these 5-HTR-DAR heterocomplexes in causing MDD development.

### 4.2. Influence of Other Heteroreceptor Complexes in MDD

#### 4.2.1. Dopamine D1R-D2R Heterocomplexes in MDD

The dopaminergic system plays a fundamental role in many physiological activities and has been implicated in numerous psychiatric disorders (see Björklund and Dunnet 2007 [151]; Klein et al. 2019 [152]). Dopamine signals through the activity of GPCRs, which according to their effects on adenylate cyclase are grouped in two different families, D1 and D2 receptor (D1R and D2R) families, depending on whether they either activate or inhibit the activity of this enzyme and on differences in their pharmacological response [153,154,155]. Furthermore, subsequent studies disclosed a multiplicity of receptors, which differed in both structure and pharmacology (reviewed by Gingrich and Caron 1993 [156], Seeman and Van Tol 1994 [155]. Thus, it was shown based on homology, pharmacology, and biochemical properties that D1R and D5R subtypes belong to the D1R family, whereas the D2R, D3R, and D4R receptor subtypes belong to the D2R family. In addition, a long and short D2R isoform has been recognized.

According to the dopamine theory, postulated by Randrup et al. (1975) [157], depression could be the result of a decreased dopaminergic function. In support of this view, both decreased dopamine cerebrospinal cord fluid levels [157,158,159] and DA turnover were found in patients suffering from depression [157,160], and many common antidepressant drugs such as dopamine agonists and dopamine reuptake inhibitors showed antidepressant activity [157,161,162,163].

However, this theory was confounded by the participation of the noradrenergic and serotonergic systems in depression, and consequently, the dopamine theory had to be considered as a part of the monoaminergic theory of depression [164,165], and as such, this theory is also unable to explain the long latency required for dopaminergic drugs to benefit depressed patients and also suffers from its lack of effectivity in a large population of depressed individuals. Nevertheless, dopamine seems to play a pivotal role in depression, now called MDD, as its effects on reward are very well known [166,167]. Its role in anhedonia, the inability to experience pleasure, has been very well established. It is considered a clinical landmark of this disorder (see above) [168,169].

Numerous dopamine heteroreceptor complexes have been described in the brain as depicted in the excellent reviews by Perreault et al. 2014 [170] and Misganaw 2021 [171], but among them, for the scope of this paper, D1R-D2R heterocomplexes, which are isoreceptor complexes as they respond to the same transmitter [172], deserve particular attention. D1R-D2R heterocomplexes are particularly found in large density in human and non-human primates in comparison with rodents, as observed with PLA and situ hybridization techniques, suggesting an evolutionary biological role for these heterocomplexes in higher CNS function [173]. D1R-D2R heteromers are predominantly elevated within the nucleus accumbens in comparison with the dorsal striatum, but they have also been described within the medial prefrontal cortex and amygdala [173]. They have also been demonstrated in samples of total protein derived from the cerebral cortex of normal and depressed individuals [174]. It should be noted that in a study examining the role of single nucleotide polymorphisms as a risk factor for depression in a population of young (7–18 year-olds) Costa Rican individuals, a significant interaction effect was found between rs1039089 and rs877138 located upstream of the DA D1 and the D2R, respectively. It suggests the possibility that an imbalance of D1R-D2R heterocomplexes may be involved in the manifestation of depressive symptoms [175].

Interestingly, although D1R and D2R signal through Gs and Gi proteins, respectively [154,176], D1R-D2R heterocomplexes activate Gα_q/11_ [175]. This leads to an enhancement of intracellular calcium, which triggers Ca-calmodulin kinase 2 phosphorylation and ultimately elicits an increase in brain-derived neurotrophic factor (BDNF) within the nucleus accumbens [177,178,179], a region highly implicated in depression [180,181,182]. Functionally, D1R-D2R heterocomplexes were found to be increased in postmortem striatal samples from patients suffering from MDD as compared with control individuals [183]. In line with this last result, as will be considered below, prefrontal cortex infusion of an interfering peptide (Tat-D2L_IL3-29-2_), which disrupts D1R-D2R heterocomplexes, produced significant antidepressant effects both in the forced swimming test [183] following its i.c.v. administration and in the 5-day learned helplessness test. However, these effects seem to be region-specific, as no antidepressant effects were noticed when the interfering peptide was infused into either hippocampus or nucleus accumbens [183]. Similar results were later confirmed by Shen et al. 2015 [184], who reported that disruption of D1R-D2R heterocomplex by a different (TAT-D1) peptide following its i.c.v. infusion prevented the depressant effects induced by D1R-D2R heterocomplex activation in the forced swimming test.

The mechanism underlying the depressant effects of the D1R-D2R heterocomplex is not clear, but it is, however, likely that both its enhancing properties on BDNF expression in the nucleus accumbens and an increase in GABA mediated inhibition in this region may participate in the mediation of these effects. In support of the BDNF involvement in this mechanism, it has been reported that this neurotrophic factor exerts, within nucleus accumbens, depressant effects [185,186], although the opposite outcome has also been demonstrated. In support of the GABA involvement in depression, it has been reported that the D1R-D2R heterocomplex can augment the accumbal expression of glutamate decarboxylase 67 (GAD67), the rate-limiting enzyme for GABA synthesis. This leads potentially to an increase in the GABAergic inhibitory tone in the nucleus accumbens and to the appearance of anhedonia and other depression-related behaviors [181,182]. It is worth mentioning in support of the role of D1R-D2R receptor heterocomplexes on depression that they are present in higher density in female rat striatum than in males and showed a higher sensitivity to the depressant effects of the D1R agonist SCH 83959 exerted on this heterocomplex. As expected, these effects were ameliorated by the heteromer disrupting peptide TAT-D1 [173].

#### 4.2.2. GalaninR2 (GalR2)-NPYY1R Heterocomplexes in MDD

NPY is a 36 amino acid neuropeptide, which was originally extracted from the brain by Tatemoto et al. [187]. It has been encountered in many brain regions [188], particularly in the hippocampus. Here it has been found in a large population of neurons within the polymorphic layer of the dentate gyrus [189]. Furthermore, in line with these findings, NPYY1 receptors are also expressed in this anatomical location [190,191]. Behaviorally, high NPY receptor signaling seems to be associated in rats and mice with decreased immobility in the Forced Swim Test, suggesting that it plays an important role in the modulation of mood [192,193,194,195,196].

Recent work by Narváez et al. [197] using receptor autoradiography, in situ hybridization, and proximity ligation assay has unveiled the likely existence of a physical interaction between NPYY1R and GalR2R in the hippocampus giving rise to the formation of putative GalR2-NPYY1R heterocomplexes within the polymorphic and subgranular subregions of the dentate gyrus. In support, it was found that the i.c.v. injection of galanin increased both NPYY1R agonist binding and NPYY1R mRNA expression in the dentate gyrus. In addition, evidence was obtained by the same group suggesting that signaling through such heterocomplexes may be important for the modulation of mood. It was demonstrated that despite the observations that galanin itself, as previously discussed, elicits anxiogenic effects, it could induce a strong enhancement of the NPYY1R antidepressant activity in the Forced Swim Test when co-administered together with an NPYY1R agonist.

It seems likely that different galanin receptors are involved with GalR2 mediating the antidepressant effects and GalR1 the depressive and anxiogenic actions [198].

#### 4.2.3. OpioidR Heterocomplexes in MDD

Experimental evidence has shown that classical μ (MOR), δ (DOR), and ᴋ (KOR) opioids are involved in MDD and anxiety [199]. Moreover, in agreement with this, it has also been recognized that aberrant MOR activity is involved in mediating social anhedonia [200], which is widely accepted as a landmark of MDD. It is also interesting that genetic and pharmacological evidence indicates that contrary to the view [201] that MOR activity triggers antidepressant and anxiolytic receptors, they play a significant role in both MDD and anxiety. Opioid receptor dysregulation may, in fact, exist at the heart of the pathophysiology of MDD [200]. Dysregulated MOR is involved in altered mood effects, and its activity may at least under certain conditions gate depressive and anxiogenic states. In agreement, it has been reported that MOR KO mice display an anxiolytic behavior in the Forced Swim Test and that Tyr-D-Ala-Gly-MePhe-Gly-ol-enkephalin (DAGO) administration, a MOR receptor agonist, results in the appearance of anxiogenic effects [202]. These results suggest that the activity of this receptor may even trigger depressive and anxiogenic states.

Experimental evidence also indicates that KOR activity seems to be endowed with depressive effects as pharmacological interferences with its neural activity lead to the appearance of antidepressant effects, as indicated by a decrease in immobility in the Forced Swim Test [203,204,205].

On the other hand, there seems to exist common agreement as to the view that DOR receptor activation is accompanied by the appearance of antidepressant effects. Thus, increased levels of immobility are observed in DOR KO mice in the Forced Swim test [206]. Furthermore, the treatment of rats with DOR agonists gives rise to antidepressant behaviors [207]. Interestingly, and in agreement, positive synergistic antidepressant-like effects have been reported following combined treatment of mice with a KOR antagonist and a DOR agonist [204]. It is, however, noteworthy that although it is generally accepted that agonist specificity for each type of opioid receptor exists, there is remarkable promiscuity among all of them. In agreement, it has been reported based on the binding and signaling properties of more than 20 different endogenous peptides belonging to all the classical opioid receptor families that all of them can bind and activate all opioid receptors. Moreover, it was even observed that some of these peptides need their activity-biased signaling favoring the engagement of a particular signaling pathway [208].

On the other hand, evidence for receptor heteromerization between MORs and DORs [209,210,211] as well as between KORs and DORs [36] has been gathered using cellular models. Most importantly, during these studies. It was revealed that because of the physical interaction between the interacting receptors, allosteric interactions take place between the two protomers. It leads to the appearance of emergent properties in ligand recognition, trafficking, and signaling, which are different from those originally present in the interacting protomers. As most of the evidence showing the existence of homo and hetero receptor complexes and their protomer interactions has been obtained in cellular models, it has been of considerable interest to study them also in the brain. Using in vivo neuronal coexpression of a double mutant delta and mu-opioid receptors (delta-eGFP and mu-cherry) [212], it was possible to demonstrate colocalization of mu-cherry and delta-eGFP) in neurons of the hippocampus and hypothalamus. As these regions have been considered of primary importance for the modulation of MDD and anxiety, this finding lends support to the involvement of DOR-MOR heterocomplexes in their receptor-receptor interactions in the modulation of MDD and anxiety.

Of particular interest are the findings showing that receptor heterodimerization does not only occur between receptors belonging to the opioid receptor family but that they also take place between them and other GPCRs belonging to different neurotransmitter systems. Thus, it has been shown that MORs can interact and form receptor heteromers with α_2A_ adrenergic receptors [213,214,215].

DORs and KORs do the same but with β_2A_ receptors [216,217,218]. Similarly, MOR heteromerization has been discovered to take place with HT1AR [219] and 5-HT2AR [220] as well as with dopamine D2R [221] and D4R [222]. For additional information on opioid receptor-GPCR oligomerization [223,224]. These results indicate a major role for multiple opioidR-monoamineR heterocomplexes in the brain by integrating signaling of opioids and monoamines in many brain networks, especially the emotional networks, including the reward and antireward networks

As far as the role of opioid receptors is concerned, DOR agonists, which have been shown to have the ability to bind and activate MOR-DOR heteromers display both antidepressant and anxiolytic effects in animal models [225,226] and positively modulate BDNF expression in rats. Furthermore, in experiments designed to understand the role of MOR-DOR heterocomplexes in MDD and anxiety, it was shown by Kabli et al. [226] that dissociation of this heterodimer by the intra-accumbal administration of an interfering peptide was able to suppress the antidepressant and anxiolytic actions induced by the DOR agonist UFP-512 (H-Dmt-Tic-NH-CH(CH2-COOH)-bid). Given the recognized participation of nucleus accumbens in the modulation of MDD and anxiety [181,182,227,228], the results of this experiment strongly supported the involvement of the MOR-DOR heterocomplex in the modulation of MDD and anxiety.

## 5. Receptor-Receptor Interactions with Relevance for Anxiety

Anxiety and depression are different clinical entities that exhibit remarkable comorbidity and show similar pharmacological characteristics, suggesting that related neurobiological mechanisms may be involved in their pathophysiology and play a role in their response to different environmental conditions and pharmacological interventions. In view of that, it is not surprising that many receptor oligomers having a prominent role in the pathophysiology and treatment of depression also fulfill an important role in anxiety.

### 5.1. 5-HT1AR-5-HT2AR Isoreceptor Complexes in Anxiety

The hippocampal localization of 5-HT1AR-5-HT2AR isocomplexes within the mouse brain, as well as the antagonistic receptor-receptor interactions, which result from the allosteric modulation between their receptor protomers, were discussed above. Although the role of 5-HT1AR-5-HT2AR isoreceptor complexes on anxiety modulation has not been studied in sufficient detail, it is known that these complexes are very sensitive to stress exposure. Thus, a critical reduction in their density was reported in the pyramidal cell layer of the dorsal hippocampus [73,229], 2 h following the acute exposure of rats to the Forced Swim Test. In line with these changes, systemic administration of the selective 5-HT1AR antagonist WAY100635 to mice that were previously exposed to a post-traumatic stress model further enhanced their already elevated anxiety levels. Moreover, an increase and a decrease were observed in the expression of the 5-HT2AR and the 5-HT1AR, respectively [84]. As high 5-HT1AR activity has been linked with anxiolytic effects [230] and 5-HT2AR activity with anxiogenic actions [231], it is feasible that the elevated anxiety levels observed in these experiments resulted from the relative activities of both 5-HT receptors favoring an enhancement of the 5-HT2AR over the 5-HT1AR activity. Such effects may reflect the existence of antagonist allosteric interactions that was reported to exist between the two protomers in the 5-HT1AR-5-HT2AR isoreceptor complex.

Future relevance for anxiety. As the serotonergic system has been demonstrated to be deeply involved in anxiety and mood disorders, and both clinical entities show a high degree of comorbidity, it will be highly desirable to find out which neurobiological aspects are in common in both clinical entities and to establish a possible role of 5-HT1AR-5-HT2AR heterocomplexes in the development of such comorbidity. As 5-HT signaling has been long involved in food intake [232] and ghrelin through its action on GHSR1AR activity is known to be at the heart of stress-mediated food intake (see below), it will be rather interesting to study whether higher-order oligomeric complexes become involved. The molecular association of 5-HT1AR, 5-HT2AR, and GHSR1a protomers does exist in the brain and may represent a bidirectional molecular link-making interactions between stress-elicited food intake and depression/anxiety possible.

### 5.2. Opioid Heteroreceptor Complexes in Anxiety

As in MDD, opioid neurotransmission plays a prominent role in anxiety. Thus, as discussed by Browne and Lucki [200], pharmacological blockade of MORs enhances the acquisition of conditioned fear and increases freezing in response to a conditioned stimulus, while KOR antagonism shows opposite effects [233]. In agreement, morphine treatment attenuates the acquisition of fear memories both in rats [234], mice [235], and humans [236]. Moreover, this effect seems to be mediated by specific brain areas such as the amygdala and the periaqueductal gray [237]. Particularly, the MOR-rich intercalated paracapsular islands are involved. Within the amygdala, they form a GABAergic inhibitory interface that surrounds the basolateral nucleus and gate nerve impulses from the basolateral nucleus (BLA) and infralimbic cortex to the neighboring central amygdaloid nucleus (CeA) [238].

In addition, it is worth mentioning that, unlike MOR-mediated effects, KOR activity seems to be endowed with anxiogenic properties, as consistently demonstrated following its pharmacological antagonism in several unconditioned models of anxiety, such as the Elevated Plus-Maze [239,240,241], the Open-Field [242], and the Defensive Burying Test [243], in which anxiolytic effects were observed. In agreement, similar results were also observed in the fear-potentiated startle, an anxiety-conditioned paradigm [240,241]. Furthermore, it was also observed that whereas both BLA and CeA were involved in the effects of KOR on fear conditioning, BLA was only responsible for their effects on anxiety [241]. In support of the aforementioned pharmacological evidence, lower levels of basal anxiety were measured in mice having ablation of KORs affecting brain dopamine neurons [244].

In turn, DOR activity exhibits an anxiolytic profile, which is widely supported by experimental results gathered in mice following ablation of one of the functional variants (DOR*1*) of this receptor, which triggers the appearance of anxiolytic effects both in the Elevated Plus-Maze and the Light-Dark Box [206] indicating that *Oprd1*-encoded receptors contribute to lowering anxiety levels and by pharmacological experiments, which revealed that whereas the DOR agonist administration triggers anxiolytic effects [245,246,247,248], DOR antagonists elicit anxiogenic behaviors [248]. Interestingly, it has been reported that an increase in anxiety levels accompanied by an enhancement of alcohol self-administration was observed in DOR knockout mice in the Light-Dark Box test in comparison with the wild-type phenotype [249]. It suggests that DOR receptors may participate in the modulation of the basal anxiogenic tone.

Future relevance for anxiety. As indicated above (see Section 4.2.3), evidence for receptor heteromerization between MORs and DORs, as well as between KORs and DORs, having relevance for anxiety and depression has already been achieved. However, as most of the evidence showing their existence and their properties has been assembled in cellular models and their existence within the brain has only been limited to some few regions such as the hippocampus, hypothalamus, and presumably the nucleus accumbens, it would be very important for both to map them out and to study their functional properties in many other regions having relevance for the modulation of anxiety, such as the amygdala, lateral septum, or the periaqueductal gray, among others. Such information will be instrumental for developing compounds able, according to their role in the modulation of anxiety, to either disrupt them or favor their existence. On the other hand, in view of the multiplicity in the binding and signaling of opioid receptors, the development of new drugs targeting specific opioid heteroreceptor complexes in a biased way would be very promising for the development of more selective, efficacious, and safer pharmacological treatments.

### 5.3. Galanin Heteroreceptor Complexes in Anxiety

Galanin-mediated neurotransmission, as in the case of mood, has an important role in the modulation of anxiety [250]. It was early reported that i.c.v. galanin (also referred here as Gal (1–29) administration elicited anxiolytic-like effects in the rat using the Vogel conflict test [251]. In contrast to these effects, galanin infusion into the amygdala resulted in the appearance of anxiogenic effects using the same behavioral paradigm [252]. To make things even more complicated, the same study reported that galanin was devoid of effects in the Elevated Plus-Maze [252]. Subsequent studies using GalR2 null mutant mice demonstrated, however, an anxiogenic-like phenotype in the Elevated Plus-Maze and other tests of anxiety [253]. Similar results were also found in the same test using GalR3 KO mice [227]. A different set of experiments was, however, useful by demonstrating that under heightened noradrenergic conditions, Gal (1–29) is released in the amygdala and can buffer the anxiogenic-like behavior elicited in the rat by the acute stress promoted by the Elevated Plus-Maze [254,255]. In addition, the anxiogenic effects of yohimbine in wild-type mice were suppressed in conditional transgenic mice overexpressing Gal (1–29) in noradrenaline neurons [256]. Thus, it seems that the effects of galanin on anxiety are complex and very much dependent upon the test, context, and mode of administration. Interestingly, despite the above data suggesting that galanin is playing an anxiolytic role in the modulation of anxiety, it was demonstrated that i.c.v. Gal (1–15) infusion to rats is endowed with the ability to trigger strong anxiogenic effects in several anxiety paradigms [109]. Thus, it was reported that following its i.c.v. Gal (1–15) administration, a significant decrease was observed in the number of entries and in the time that the rats spent in the central square of the Open Field Test, as well as a significant decrease in both the total time spent in the light compartment and in the latency to enter into the dark chamber of the light/dark test apparatus as compared to their respective controls in both tests [109]. On the other hand, as Gal (1–15) has been shown to preferentially bind to the GalR1-GalR2 heteromeric complexes in comparison to Gal (1–29) [2], it seems reasonable to propose that the anxiogenic effects of this Gal (1–29) fragment result from the activity of GalR1-GalR2 heteroreceptor complex in which anxiogenic rather than anxiolytic actions are developed. In support of this suggestion, besides the fact that BRET and PLA assays have shown the existence of this heteroreceptor complex both in HEK293T cells and the brain, respectively [2,257], it was revealed that the blockade of the GalR2 receptor prevented the effects of the Gal (1–15) [109]. In line with these results, siRNA GalR2 knockdown mice failed to behaviorally respond to Gal (1–15) fragment administration in the same anxiety models [109]. It lends additional support to the suggestion that Gal (1–15) is recognized and exerts its effects through GalR1-GalR2 heterocomplex.

Future relevance for anxiety. As specified above (see Section 4.1.3), the effect of galanin1-15 on anxiety seems to a large extent to be dependent upon its interactions with the serotonergic system through its effects on GalR1-GalR2-5-HT1AR heteroreceptor complexes. As the strongly anxiogenic Gal (1–15) fragment binds to the GalR1-GalR2 heterodimer, it will be of interest, as in the case of depression (see above), to clarify the role of Gal (1–29) vs. the galanin fragment (1–15) in the modulation of the GalR1-GalR2-5-HT1A and GalR1-GalR2 heterocomplexes. Furthermore, it will be of interest to elucidate which are the factors involved in the biochemical relationship between Gal (1–15) and Gal (1–29). It will also be important to know whether GalR1-GalR2-5-HT1A heterocomplexes exist in the amygdala and other brain regions known to participate in the modulation of anxiety.

### 5.4. Gal-NPY Heteroreceptor Complex in Anxiety

NPY, in addition to exhibiting antidepressant properties, has also been reported to display important anxiolytic effects in different behavioral models [250,258,259,260]. It has also been reported that such effects are mediated through the activity of one of its receptor subtypes, the Y1 receptor subtype [251,261,262]. In addition, recent studies employing i.c.v. administration have shown that NPYY1 and GalR2R receptor coactivation results in anxiolytic effects in different innate tests of anxiety, which include the Open-Field, the Elevated Plus-Maze, and the Light-Dark Box. In this way, the agonist-induced NPYY1R anxiolytic activity was significantly enhanced due to the GalR2 agonist coactivation. These results suggest the presence of important interactions between Gal (1–29) and NPY signaling systems as they are not dependent on locomotion, and the enhancing effects induced by GalR2 coactivation upon NPYY1R activity were counteracted by M871, a GalR2 antagonist [260]. Highly important for the scope of this work is the observation that, along with the behavioral changes described above, an increase in the density of GalR2-NPYY1R hetero complexes also was observed. Such results were also unveiled within the amygdala and were specifically occurring within the dorsolateral intercalated islands but not in the ventromedial intercalated islands [260]. It is known that intercalated islands form an inhibitory GABAergic interphase between CeA and BLA (see for a review Palomares-Castillo et al. [238]) and interact in a dorsal-ventral position with one another in a complex manner. Dorsally located intercalated islands inhibit the ventrally located insular component [263]. On this scenery, it may be feasible that activity in GalR2-NPYY1R heteroreceptor complexes, which, as indicated above, are elevated upon agonist coactivation of GalR2 and NPYY1R [260], may allow for an increased inhibitory tone upon CeA neurons leading to a reduction in the anxiogenic CeA output with the consequent diminution of anxiety. In support of this view, a decrease in C-Fos expression, a marker of neuronal activity, was reported within the ventromedial intercalated islands following the coactivation of NPY Y1R and GalR2. As a corollary, under those conditions, C-Fos expression was also found diminished within the ventromedial hypothalamic region and the ventrolateral part of the periaqueductal gray, which receive CeA afferents and are known to be involved in the anxious response [264,265].

Future relevance for anxiety. GAlR-NPYY1R heterocomplexes have been demonstrated within the dorsolateral component of the intercalated amygdaloid islands (see above) and there is evidence suggesting the presence of MOR containing heterocomplexes in such islands (see Section 5.2). As neurotransmission between islands, as discussed in Section 5.2, is polarized in such a way that dorsally located islands keep in check ventrally located islands, it will be of considerable interest to know whether MOR containing heteroreceptor complexes, in fact, exist within these islands and if so their topography.

### 5.5. Dopamine D1R-D2R Heterocomplexes in Anxiety

Dopamine signaling has a paramount importance in the pathophysiology of anxiety, as demonstrated in conditioned and unconditioned models of this disorder, and its role in this matter has been extensively reviewed by Pezze [266]; Pérez de la Mora et al. [267]; Zarrindast and Khakpai. [268] Pérez de la Mora et al. [269]). The experimental evidence provided in those studies suggests that DA D1 receptor signaling consistently triggers anxiogenic responses, both in conditioned and unconditioned anxiety models [270,271,272,273,274]. In contrast, the effects of DA D2 receptor signaling have been found to be substantially dependent upon the paradigm used [268,275,276].

On the other hand, the existence of dopamine D1R-D2R heteroreceptor complexes has been demonstrated in the amygdala and hippocampus [173], which are generally accepted to play a critical role in anxiety modulation [238,266,267,277,278,279]. Dopamine D1R-D2R heterocomplexes have been mostly studied, as already discussed, in the context of MDD. However, according to the studies carried out by Susan R George and collaborators, D1R-D2R heterocomplexes also seem to have a significant role in the pathophysiology of anxiety. Agonist stimulation of D1R-D2R heterocomplexes elicits, in rats, anxiogenic effects in the Elevated Plus-Maze as indicated by the observed D1R-D2R agonist SKF 83959-induced reduction in both the time spent in as well as the number of entries into the open arms of the maze [184]. In keeping with these effects, SKF 83959 treated rats experienced both an increased latency to drink milk and markedly reduced their consumption of milk, as compared to control animals, when they were tested in the Novelty Suppressed Feeding Test [184], which are considered to represent anxiety measures in this paradigm [280]. As expected from the putative ability of D1R-D2R heterocomplexes to mediate the anxiogenic effects of SKF 83959, i.c.v. pretreatment with the D1R-D2R heterocomplex disrupting peptide TAT-D1, significantly suppressed or attenuated those effects [184]. Moreover, the observation that in those experiments, TAT-D1 peptide by itself was able to elicit anxiolytic effects suggests that D1R-D2R heterocomplexes may be at least to a certain extent constitutively active within the brain. As may be expected from the effects of the agonist-stimulation of D1R-D2R heterocomplexes in MDD in female rats (see above), anxiety behavior was more prominent in them than in males and was also ameliorated by the disrupting effects of the TAT-D1 peptide administration [173].

Future relevance for anxiety. Given the anxiogenic role played by activation of the D1R-D2R heterocomplexes and the amelioration of these effects upon its disruption with a TAT-D1 peptide, it will be highly relevant to pursue this promising line of research by developing new disrupting peptides having smaller sizes and improved brain permeability. Alternatively, the search for bitopic compounds should also be considered. D1R protomer activation always results in anxiogenic effects, but effects upon D2R activation seem to depend on the model used. Approaches to finding biased ligands that may stimulate different signaling pathways with separate roles in anxiety may also be considered. Finally, as D1R-D2R heterocomplexes seem to exist constitutively in parts of the limbic brain, studies on their contribution to maintaining basal activity are warranted.

### 5.6. D2_L_R-5-HT1AR Heterocomplexes in Anxiety

As described above, D2_L_R-5 HT1AR hetero complexes have been described both in cellular models and within the dorsal raphe nucleus [149,150]. Such receptor heterocomplexes have been shown, as in the case of MDD, to also play an important role in anxiety. Thus, an anxiogenic profile in both the Open Field Test and the Elevated Plus-Maze was observed in D_2L_ KO mice. It was demonstrated that a dysfunction of the 5-HT1AR developed under these conditions in the 5-HT neurons associated with stress vulnerability. It was proposed that the D2_L_R-5-HT1AR heterocomplex may function as an inhibitory feedback modulator to suppress psychosocial stress [149].

### 5.7. D2R-OXTR Heterocomplexes in Anxiety

Unlike other neurotransmitter receptor systems, a single type of oxytocin receptor (OXTR) has been found to be responsible for mediating all actions of oxytocin both in the periphery and brain (see the excellent and comprehensive review on this topic by Jurek and Neumann [281]). Thus, among other neural functions, oxytocin neurotransmission has been critically implicated in the modulation of anxiety [281,282] through its participation as a transmitter within the amygdala [283,284,285,286,287].

The recent work from our laboratory using the Shock-Probe Burying test [64] is in close agreement with the anxiolytic effects of oxytocin on the amygdaloid modulation of anxiety discovered by Bale and associates in the Open Field Test [285] and by Blume et al. [288] in both the Light-Dark Test and the Elevated Plus-Maze. Our results also agree with the anxiolytic effects of oxytocin within the amygdala reported by Laszlo et al. [289,290], using the Elevated Plus-Maze. Our work also pointed to the existence of significant functional interactions between oxytocinergic and dopaminergic D2R-mediated signaling within the amygdala [64] as the anxiolytic effects observed following the intra-amygdaloid administration of oxytocin in the Shock Burying Test were abolished by the blockade of dopamine D2 receptors elicited by the infusion of raclopride within the amygdala [64]. Moreover, these findings suggest the existence of a close anatomical interaction between both neurotransmitter systems as they were demonstrated following the simultaneous infusion of both compounds in the CeA amygdaloid nucleus [64].

D2R-OXTR heterocomplexes possessed facilitatory allosteric receptor-receptor interactions and were demonstrated by PLA in both ventral and dorsal striatum [291] (Figure 1). They were also identified in HEK293 cells following transfection of both receptors and the coexistence of D2R and OXT receptors is clearly evident in CeA. It may be possible that the receptor-receptor interactions described above may have taken place within intra-CeA D2R-OXTR heterocomplexes. The blockade of facilitatory protomer interactions by raclopride within D2R-OXTR heterocomplexes suppressed the anxiolytic signaling induced by oxytocin in these heteroreceptor complexes. Interestingly and in support of these studies in HEK293 cells [64], results indicate that reciprocal protomer interactions between D2 and OT receptor signaling do exist within these D2R-OXTR heterocomplexes. They enhance the coupling of their respective signaling cascades and thereby the anxiolytic effects of oxytocin. Thus, it was demonstrated using HEK293 transfected cells that oxytocin increased the potency of the D2 receptor agonist quinpirole to inhibit the activity of the AC-PKA-CREB pathway through enhancing Gαi/o-protein coupling. Likewise, quinpirole enhanced the oxytocin protomer signaling over the Gα_q/11_ G protein-mediated PLCβ-IP3-calcineurin pathway. As a corollary of these results, both quinpirole and oxytocin had the ability to enhance the RAS-MAP-pELK pathway via activity in their respective βϒG-protein dimers.

Future relevance for anxiety. In view of the pharmacological evidence that supports the presence of anxiolytic D2R-OXR interactions within the amygdaloid central nucleus and that the occurrence of D2R-OXR heterorcomplexes has been unveiled within the nucleus accumbens, it should also be of considerable interest to study whether such receptor heteromers are also present in the amygdala. It will also be of importance to understand the mechanism involved in the presumed anxiolytic effects produced by the D2R-OXR heterocomplexes and to identify the precise topographical location of these heteroreceptor complexes within the amygdala. In consideration of the anxiolytic effects attributed to the activity of the D2R-OXR heterorcomplexes, efforts to design either heterobivalent or bitopic compounds aimed to favor their anxiolytic effects should be attempted.

## 6. Ghrelin

### 6.1. Growth Hormone Secretagogue Receptor 1a (GHSR1a)-Containing Heteromer Complexes as Links between Stress-Induced Eating Behavior with Depression/Anxiety

Unlike protomers from other heteroreceptor complexes, growth hormone secretagogue receptor 1a (GHSR1a) behaves like a special protomer, which through its binding to ghrelin is related, on the one hand, to the homeostatic control of food intake and energy expenditure and via its allosteric interactions with several other protomers (i.e., D1R or 5-HT2CR), influences mood and emotion.

Ghrelin is a 28–amino acid peptide derived from the cleavage of a prepropeptide having 117 residues [292,293], which has a major influence on the modulation of appetite and food intake [294] but also has been linked to depression, stress, and anxiety [292,295], most probably due to its well-known orexigenic effects. In keeping with this, numerous studies have demonstrated a close relationship between obesity and psychiatric disorders [294,296,297] and predominantly with depression [298,299] and anxiety [300,301,302].

Ghrelin, and particularly its acylated form, binds with high affinity to the growth hormone secretagogue receptor 1a GHSR1 [303,304,305], triggering a complex signaling process [306], resulting in an increase in endogenous calcium mobilization mediated through a Gαq protein activation [307]. In addition, arrestin2-dependent mechanisms on trafficking and other signaling cascades have also been recruited upon GHSR1 activation [308].

In situ hybridization studies have reported that mRNA encoding GHSR1a is largely expressed in the brain [295,309,310], where it has been particularly found in the ventral tegmental area (VTA) and some limbic regions such as the hippocampus, nucleus accumbens, and amygdala, which are known to be linked to modulation of depression and anxiety (see above). Evidence for brain ghrelin sources is scarce and peripherally-born ghrelin only seems to reach, in substantial amounts, the nucleus arcuate in the hypothalamus [311], from where it may exert its orexigenic effects. However, it has been suggested that ghrelin may reach other brain regions via the ventricular brain system following its entry into the brain through the blood vessels that irrigate the median eminence [311]. Additionally, splice variants of the ghrelin gene may also be produced, having the capacity to stimulate GHSR1a through their peptide products [312]. It is, however, worth underlining that despite its high affinity for ghrelin, GHSR1a also is endowed with a high constitutive activity, which reaches 50% of its maximal activity in the absence of ghrelin [313,314] which may be modulated by both modifications in its expression and by allosteric influences triggered by its association with other neurotransmitter receptors within heteroreceptor complexes.

In view of the above, it may be reasonable to think that ghrelin is mediating a bidirectional link between food intake and depression/anxiety through the promiscuous interaction of GHSR1a with other neurotransmitter receptors within a variety of heteromereceptor complexes such as GHSR1a-D1 [307,315,316]. GHSR1A-D2 [317], GHSR1A-MC_3_ [307,315,318], and OXTR-GHSR1a [319].

### 6.2. GHSR1a-DA Receptor Heteroreceptor Complexes

Palatable high-caloric food ingestion is known to ameliorate the effects of chronic stress [320], as well as those effects of ghrelin promoting food intake [321,322]. Furthermore, ghrelin has been genuinely considered a guard, which protects against the deleterious effects of chronic stress [310,323]. Although the effects of centrally or peripherally administered ghrelin on mood and anxiety have so far been rather contradictory and to be dependent on many variables such as the animal’s previous stress conditions, their feeding state (Alvarez-Crespo et al. or the behavioral paradigm used [321] many results, which suggests a protective role of ghrelin against depression and anxiety, which is exerted by its binding to GHSR1a under chronic stress conditions [321,323]. In line with these results, DNA recombinant studies have shown that following stress exposure, GHSR1 KO mice exhibit stress coping deficits confirming the protective role mediated by the activity of GHSR1a [321]. On the other hand, it is well known that the dopaminergic mesolimbic system is critically involved in different aspects of the food reward system [324,325], but that also plays a pivotal role in the development of obesity [324,325], depression [168,181,326], and anxiety [266,267,268,269].

### 6.3. GHSR1a-D1R Heteromers

It is of considerable interest that evidence for colocalization of GHSR1a and D1R has been found in HEK293A cells [307,316], which, together with the agonist-induced cointernalization of both receptors discovered in the same cell model [307], suggests the existence of GHSR1a-D1R heteromers. Coimmunoprecipitation of D1R and Xpress-tagged GHSR1 from cell lysates of HEK293 cells expressing both markers [316] together with studies of bioluminescence resonance energy transfer (BRET), which, as indicated above, allows the identification of proteins lying in close proximity, confirmed the putative existence of GHSR1a-D1R heteromers. In agreement with these results, real-time single-molecule analysis on hippocampal brain slices has further established its existence within hippocampal neurons and provided evidence for its dependence on D1R as GHSR1a-D1R heteromer formation was enhanced by SKF81297, a D1R receptor agonist [314].

From a functional point of view, it has been revealed [316] that GHSR1a, as a consequence of its protomeric interaction with D1R within GHSR1a-D1R heteroreceptor complexes, switches its signaling from Gα_q/11_ to G _i/0_ leading to a potentiation of the dopamine-induced cAMP accumulation. Such potentiation requires both receptors and both ligands [316], and it is suggested that the Gβγ dimer associated with GHSR1a acquires a stimulatory role in the adenylyl cyclase activity [316], leading to an amplification of the dopamine D1R signaling. In contrast to the latter effects, it has also been shown, using the same model, that following D1R and GHSR1a dimerization, the increase in intracellular calcium resulting from the ghrelin activation of GHSR1a is attenuated in HEK293A cells coexpressing D1R [307]. In addition, it has been reported that in hippocampal slices, protomeric D1R activation led to calcium mobilization from the endoplasmic reticulum through a non-canonical signaling pathway involving Gα_q_-PLC-IP_3_-Ca^2+^ leading to CaMKII activation [317] and eventually to an enhancement of synaptic plasticity as a consequence of increased NMDA currents [317].

As hippocampal-mediated plasticity has been related to depression, the above results suggest that cautious targeting of GHSR1a-D1R heteromers may be beneficial for treating mood disorders. Additionally, of particular importance for the aim of this work is the finding that JMV2959, a GHSR1a antagonist, when is simultaneously injected with SKF81297 into the hippocampus, prevented the D1R-induced extinction memory consolidation [317], signifying that GHSR1a protomers may have a facilitating action on the modulation of anxiety through their allosteric interactions with D1R protomers within GHSR1-D1R localized within the hippocampus and perhaps in the amygdala and other brain regions.

### 6.4. GHSR1a-D2R Heteromers

The properties and function of this heteroreceptor complex have been essentially and elegantly studied by Kern and colleagues [317]. Unlike their studies on GHSR1a-D1R heteromers in which coexpression of GSHD1Ra and D2R was found in the hippocampus (see above), in this study, coexpression of GHSR1a and D2R proteins was detected in the hypothalamus despite D2R being preferentially expressed in the striatum [317]. Furthermore, time-resolved fluorescence energy transfer (tr-FRET) analysis showed that GHSR1a and D2R were lying very close to each other within a relative distance of 50–60 Å, strongly suggesting the existence of GHSR1a-D2R heteromers in this brain region [317]. Most importantly, consistent with both the formation of GHSR1a-D2R heteromers and allosteric interaction between their constituent protomers, modification of the canonical signaling properties of the D2R protomer was found upon dimerization. Thus, canonical D2R signal transduction involving adenylyl cyclase inhibition and cAMP reduction was shown to shift to an IP_3_-mediated rise of calcium mobilization due to the allosteric interaction between protomers in GHSR1a-D2R heteromers [135,327].

From a physiological point of view, since, as indicated above, GHSR1a receptors play an important role in the modulation of food intake and in its rewarding influences, the role of this heteroreceptor complex has only been considered in the context of obesity. However, as the hypothalamus, as a part of the limbic system, plays an important role in the modulation of mood and emotion, it would be highly desirable that future studies aimed to evaluate its direct or indirect participation in depression and anxiety will be carried out.

Future relevance for MDD and anxiety. Unlike the other heteroreceptor complexes considered in this work, which have a well-defined receptor protomer composition in terms of the nature of their respective neurotransmitter ligands, GHSR1a-containing heteroreceptor complexes are endowed with high constitutive activity, dimerize with a number of different protomers, and display complex signaling pathways. On the one hand, they seem to represent a promising gate through which it may be possible to study the complex relationship between stress-induced eating behavior with depression and anxiety. On the other hand, GHSR1a, because of the “promiscuity” of its interactions with a variety of neurotransmitter receptors (protomers) and signaling pathways, offers more than any other protomer the unique possibility of fine-tuning specific protomer partners in region-specific heteromers involved in the modulation of depression and anxiety with a reduced side-effect profile.

As the expression of ghrelin within the brain is rather controversial and it is known that GHS-R1a is endowed with high constitutive activity, it would be highly desirable to investigate the presence within the brain of alternative endogenous ligands that could be responsible for such activity. It will also be of interest to study the role played by GHS-R1a as an allosteric modulator of other receptor protomers different from D2R, as proposed by Kern.

## 7. Novel Therapeutic Strategies for Treatment of MDD and Anxiety Based on GPCR Heteroreceptor Complexes

It is thought that between 19,000–20,000 protein-coding genes exist in the human genome [328]. Recent estimates indicate that among them, there exist at least 800 genes that codify for GPCRs [329,330,331], making them the largest family of membrane receptor proteins in the human genome. Nearly half of those genes could represent potential drug targets to mitigate human diseases [332,333]. It is interesting that GPCRs, through forming homoreceptor complexes or heteroreceptor complexes with distinct specific pharmacological profiles, can be exploited for drug design, opening new therapeutic alternatives. Particularly appealing are biased therapeutic options in which drug activation or inhibition of selective GPCRs, either through G-protein coupling or β-arrestins, may minimize, at least to some extent, the adverse effects that plague the use of conventional pharmacological medications [334,335].

On the other hand, as facilitatory or inhibitory allosteric interactions between protomers in specific heteroreceptor complexes lie at the heart of the GPCR heteromerization concept (see above), therapeutic drug developments based on cross-talk between protomers belonging to specific heteroreceptor complexes can take place. In support of this contention, it has been revealed (see above) that D2 receptor activity within presumed amygdaloid D2R-OTR heterocomplexes is involved in the anxiolytic effects of oxytocin [64]. The possibility of developing therapeutic approaches targeting these putative heteroreceptor complexes is rather appealing.

On the other hand, Gal (1–15), as discussed above [134], can enhance the antidepressant effects induced by activation of the 5-HT1AR protomer, most probably through the formation of Gal1R-Gal2R-5-HT1AR heterocomplexes. This complex upon Gal (1–15) treatment also can improve the antidepressant effects of fluoxetine and escitalopram [130]. These results suggest that combined treatment of fluoxetine and intranasal administered Gal (1–15) may offer new targets for the treatment of MDD [327] [135,327]

Another example is interactions between DOR and MOR protomers in DOR-MOR heterocomplexes occurring at the spinal cord level [209,210,211], which has been demonstrated to result in an increase in nociception [210]. Yet, it is interesting to note that colocalization of mu-cherry and delta-eGFP has been shown within hippocampal and hypothalamic neurons [212], suggesting the existence of protomeric cross-talk in DOR-MOR heterocomplexes has relevance for MDD and anxiety. This complex is likely susceptible to a therapeutic approach.

Furthermore, antagonistic allosteric receptor-receptor interactions in A2AR-D2R heterocomplexes [38,136] within A2R-D2R receptor heteromers [136,327,336,337] have been instrumental in developing promising A2AR antagonists aimed at treating motor dysfunction and potentially neurodegeneration in Parkinsonian’s patients [94,338,339,340]. A2AR agonist targeting the A2AR-D2R heterocomplexes in the limbic system leading to inhibition of D2R protomer signaling offers a novel treatment for patients with schizophrenia. The major target for the A2AR agonist should be the activation of the A2AR protomer in the A2AR-D2R heterocomplex to reduce hyperactivity in the D2R protomer in the A2AR-D2R heterocomplex in the ventral-striatal-pallidal antireward GABA pathway. In this way, the exaggerated inhibition of this pathway produced by the D2R becomes properly reduced, restoring the activity of these neurons. This activity is needed to allow an appropriate gating of incoming stimuli reducing the positive symptoms of schizophrenia [95,198,341].

These examples give an improved understanding of the important actions, the distinct recognition, signaling, and trafficking properties triggered upon GPCR heteromerization. Based on these mechanisms, therapeutic strategies can be developed to treat several psychiatric disorders by either stabilizing convenient heteroreceptor complexes or to disrupt harmful constitutive heteroreceptor complexes that have developed. Accordingly, hetero-bivalent compounds have two different pharmacophores connected by a spacer of variable length, which simultaneously target the orthostatic binding sites of both protomers in a given homo/heterodimer [342,343,344,345,346,347,348] have been synthesized. In this way, designing non-constitutive receptor heteromeric compounds will give distinctive pharmacological properties but lack the pharmacokinetic drawbacks associated with the simultaneous administration of two different compounds, which can be achieved through the use of bivalent ligands [204,342]. It also offers the possibility to either abolish or minimize the side-effect profile of the participating protomers [345,349]. Furthermore, it has also been observed that, at least for some bivalent ligands, biased signaling properties do indeed seem to exist. In fact, in cellular models, it has been shown that specific bivalent ligands acting at D2R-NTS1R heterocomplexes are capable of triggering preferential β-arrestin-2 activation [350].

As would be expected from the foregoing discussion, numerous bivalent ligands having relevant pharmacological properties have been successfully synthesized [344,346,348]. Among those compounds, it has been reported that in mice i.c.v. infusion of MDAN 21, a bivalent ligand, which bridges MOR and KOR protomers within a KOR-MOR heterodimer, is 50-fold more potent than morphine to relieve pain and is also devoid of tolerance and physical dependence following its chronic administration [351]. Following the same line, MMG22, another bivalent ligand targeting the MOR-mGluR5R heterodimer within the spinal cord, has been shown to have the ability to produce potent anti-nociception and reduce neuropathic pain in mice [5]. Likewise, homo-bivalent ligands, developed based on the use of the atypical antipsychotic drug clozapine as a pharmacophore, have been shown to induce a substantial increase in its affinity for D2 receptors. It was synthesized by McRobb et al. [352]. Unfortunately, despite the strong experimental evidence supporting the participation of receptor heteromerization in mediating numerous neurotransmitter interactions with relevance for anxiety and MDD [64], bivalent ligands of relevance for MDD and anxiety still remain to be developed.

Alternatively, peptides synthesized based on the structural information gathered on the interface between interacting receptor protomers in specific heteroreceptor complexes aimed to disrupt constitutive heteroreceptor complexes have also given promising results for the treatment of psychiatric diseases, including MDD and anxiety. Thus, prefrontal cortical infusion of a TAT-D2 peptide having the D2L_IL3-29-2_ sequence was able to disrupt D1R-D2R hetero- complexes, as measured by coimmunoprecipitation and to elicit antidepressant effects both in the Forced Swimming Test and 5-Day Learned Helplessness paradigm [183]. Moreover, it was of considerable interest that the disrupting effects of this peptide were region-specific as no effects in the Forced Swimming Test were observed by the same workers when the disrupting peptide was infused into the hippocampus or nucleus accumbens. It supports the notion that heteromer formation is region-specific [183]. In line with these observations, a different TAT-peptide (TAT-D1 peptide) designed to disrupt interactions between adjacent glutamate residues present in the D1 receptor carboxyl tail with their amino acid counterparts in the D2 receptor was also effective in uncoupling D1R-D2R heterocomplexes as revealed by both coimmunoprecipitation and BRET analysis [353]. As expected from the depressant role played by the activity of D1R-D2R heterocomplexes (see above) i.c.v. TAT-D1 peptide treatment to rats exposed to chronic unpredictable stress was able to trigger not only antidepressant but anxiolytic effects as well [184]. Furthermore, in support of the potential therapeutic approach of using D1R-D2R heterodimer disruption for treating depressive/anxiety disorders, it was also reported [184] that prior i.c.v administration of this peptide prevented the depressive/anxiogenic effects that are observed following the SKF83859-mediated D1R-D2R heterodimer formation [184]. In agreement with the above, EAARRAQE, an eight amino acid peptide derived from the third intracellular loop of the D2 receptor, has also been shown to block to the same extent the formation of D1R-D2R heterodimer both in vitro and in the temporal and in the frontal lobe tissue total protein, suggesting that no adapter/scaffolding proteins are needed for the formation of this receptor heterodimer [174]. Finally, as a corollary for the development of a successfully preclinical pharmacological treatment, a proper administration route for the recommended medications also needs to be considered. It is particularly interesting that following the intranasal TAT-D1R peptide administration described above [183], the peptide reaches the brain and exerts anxiolytic effects comparable to those exerted by imipramine [200].

It is clear that many potential GPCR targets can successfully be used to treat or at least mitigate numerous diseases. The encouraging perspectives so far attained from preclinical studies show the utility of bivalent bitopic compounds, which simultaneously target orthosteric and allosteric sites [327,354] and can be designed to promote region-specific effects. The use of bivalent ligands to either interfere with or stabilize specific receptor oligomeric conformations is also of high interest. These approaches give the possibility to obtain proper pharmacological treatment for MDD and anxiety and are promising (Table 1).

### Potential Allosteric Downstream-Mediated Receptor-Receptor Interactions in the Action of Fast Antidepressant Effects of Ketamine

Ketamine, a racemic mixture containing equal parts of (R)-ketamine and (S)-ketamine enantiomers, is a well-known anesthetic compound that is known to block NMDA receptors [357]. Ketamine has recently attracted attention for its promising antidepressant properties [358], which unlike the traditional monoamine-based treatments [21], exerts its beneficial effects in a matter of hours following its administration [21,359,360,361,362,363,364,365]. Although the way ketamine elicits its antidepressant effects is not entirely clear, it has been suggested that its effects may involve the blockade of NMDA receptors at inhibitory GABA neurons within the medial prefrontal cortex and hippocampus, leading to an increase in glutamate release [362,366]. In addition, although still controversial [367], glutamate α-amino-3-hydroxi-5-methyloxazole-4-propionic acid (AMPA) receptors also seem to have a role in the ketamine-induced antidepressant effects and have been advocated to explain the ability of AMPA receptors antagonists to block the antidepressant effects of ketamine in rodents [362,366,368,369].

An enhancement of brain-derived neurotrophic factor (BNDF) action and its associated increasing effects in both hippocampal and prefrontal cortex neurogenesis has also been suggested to explain the antidepressant effects of ketamine [366,367,370,371,372,373]. Accordingly, a link between ketamine-induced antidepressant effects with an increase in BDNF levels was reported in the rat hippocampus [374], and in full agreement with this finding, it was also shown that ketamine treatment failed to elicit antidepressant effects in BDNF knockout mice [373,375]. There are findings strongly indicating that antidepressant drugs act directly by binding to TRKB receptors [376]. Furthermore, in the clinical setting, peripheral BDNF levels in depressed patients correlated with the efficacy of the ketamine treatment, being higher in those patients who responded to the ketamine treatment [377,378]. Interestingly, although mTORC1 signaling activity lies downstream of the BDNF-TrkB cascade [379], it was thought mTORC1 mediated the antidepressant effects of ketamine, but its involvement in this mechanism has recently been challenged. Hence, although it was originally shown that its inhibition in the rat was able to antagonize the antidepressant effects of ketamine [371], recent studies have revealed that those effects only were valid for the (R)-ketamine enantiomer, which, as indicated above, holds the most powerful and longest antidepressant ketamine properties [135,367]. In particular, a very recent report shows that in depressed patients, oral treatment with rapamycin (a TORC1 inhibitor) rather than preventing the antidepressant ketamine effects potentiates them [380]; the discovery that TrKB activity and signaling can be modulated through its allosteric interactions with different receptors, such as the p75 neurotrophin receptor [381,382,383], opens up the likelihood that the TrKB receptor may also be influenced by several other neurotransmitter receptors like DA, 5-HT and adenosine receptors [75]. As a signaling alternative, it has also been proposed, based on the inhibitory effects of SL327, an ERK inhibitor, that activation of this factor may also be involved in the antidepressant effects of (R)-ketamine [384].

It is of special interest that ketamine can have direct effects on the 5-HT2R and D2R, as observed in 2002 [385]. It will be of special interest to understand if the D2R-5-HT2A heterocomplex is especially vulnerable to ketamine treatment [131,135,138]. Presently the relevance of the high affinities of ketamine for D2R and 5-HT2R is not clear regarding the role of ketamine in mental disorders. In 2002, it was also found in clinically relevant concentrations that ketamine can bind to M1 and M3 receptors in cell lines, but functional consequences could not be established [386]. In 2013, it was found that ketamine can also bind to the opioid receptor [387]. It is of substantial interest that molecular recognition of ketamine takes place through a subset of olfactory GPCRs that can function as targets for ketamine [388]. These findings have inspired us to study if ketamine can modulate heteroreceptor complexes containing one or two of the receptor protomers discussed above to understand the mechanism for the therapeutic effects of ketamine in mental disease [138].

It is of the highest interest that Saarelainen et al. [389] demonstrated that the positive effects of antidepressant drugs such as ketamine were counteracted by blocking TrkB signaling. Castren and Antila, therefore, proposed that the antidepressant effects were produced by TrkB-induced increases in neuronal plasticity [376]. The first TrkB heteroreceptor complex was recently described, with the A2AR being the other protomer (TrkB-A2AR) [75], supporting the relevance of allosteric receptor–receptor for in neuronal plasticity.

The recent work of Casarotto et al. [376] is also highly interesting as they found that ketamine can bind to the TrkB receptors in concentrations that do not block the NMDA receptor. These exciting results suggest that the rapidly acting antidepressant drug ketamine can produce therapeutic effects by binding and acting in the transmembrane domain of the TrkB receptor in limbic regions. The more rapid antidepressant actions of ketamine vs. other antidepressant drugs may possibly be explained through modulation of signaling in GPCR heteroreceptor signaling, also including NMDAR protomers.

## 8. Conclusions

It is recognized that GPCR heteromerization does, in fact, exist and that GPRs exert to a high degree their effects as homodimers, heterodimers, and high-order hetero receptor complexes. These have changed our way of understanding synaptic and volume transmission and its molecular integration. It has paved our way to transit into a new world full of surprises and unexpected receptor-receptor interactions. It will allow us to develop novel therapeutic strategies having a low side-effect spectrum and to cure or at least mitigate many psychiatric diseases such as MDD and anxiety. Convincing evidence has shown that upon heteromerization, emergent properties in terms of recognition, trafficking, and signaling develop differently from those present in the participating protomers alone. It involves allosteric and phosphorylation mechanisms to create dynamic changes in the function of the participating receptor and non-receptor protomers and the development of novel pharmacology. Thus, in pursuit of developing novel treatments for MDD and anxiety, there is a need to understand which heteroreceptor complexes are the most vulnerable to these diseases. Their dysfunction and altered balance with other hetero- and homo-receptor complexes can guide us towards novel treatments. It will be important to find drugs that also can overcome the blood–brain barrier in addition to restoring the key functions of the most vulnerable receptor complexes in MDD and anxiety.

## Figures and Tables

**Table 1:** Examples of treatment strategies related to GPCR heteroreceptor complexes.

Treatment Strategies Related to Heteroreceptor Complexes				
Strategy	Outcome	Putative Heteroreceptor Examples	Disorder	Reference
Selective protomer ligands	Induced oligomer formation	D2R-OXTR	Anxiety	[64]
		Gal1R-Gal2R-5-HT1AR	Depression and anxiety	[40,110,130,135,229,355]
		DOR-MOR	Depression, anxiety, and nociception	[205,206,207,208]
		A2AR-D2R	Parkinson’s disease and schizophrenia	[75,90,95,198,338,339,340,341]
Biased ligands	Cross-talk effects between protomers			[103,334,335,356]
Homo-/Hetero-receptor bivalent compounds	Facilitatory/inhibitory effects by binding on both protomeric recognition sites	KOR-MOR	Nociception	[351]
		MOR-mGluR5R	Nociception	[5]
Disrupting homo/heteroreceptor peptides	Disruption of constitutive heteroreceptor complexes	D1R-D2R	Depression and anxiety	[183,184,200]

## Data Availability

Not applicable.
